# The Closing of the Year

**Published:** 1891-12

**Authors:** 


### THE CLOSING OF THE YEAR.

The present number closes the twelfth volume of this journal, and before another issue the year will have left its record for 1891.

The past twelve months have been fruitful with progress, not perhaps visible to the casual observer, but clearly indicated to those who measure advances by the silent but deep undercurrent of active thought.

Professional education in dentistry received its earliest impulse in the past, but its progress has been so slow as to be almost discouraging. It was reserved for 1891 to show that the marked advance in educational work, so long contemplated, was not productive of the serious upheaval it was supposed would result; but has added stability to the colleges. That while these will, doubtless, have diminished incomes, the change has been accomplished with less friction than was anticipated, while at the same time the expansion of professional culture by the movement promises to be beyond the calculations of the most experienced.

The same inspiriting influence that led to the adoption of a higher standard in college training has also led to dissatisfaction with methods in the associations. The spirit of unrest, seeking to discard old ideas not adapted to the change in modern thought, has prevailed, and is asserting itself with a positive power in these organizations. This is the silent effect of a higher education culminating in active efforts for improvement.

There has been also an observed advance in the practical branches. Mechanical, or, as some prefer to call it, prosthetic, dentistry has resumed its proper place from which it was dethroned by the plastic bases, and promises to take a position as a specialty in dentistry. This tendency to segregation has developed rapidly within the past year. It may seem singular to some of the older workers to have the statement made that dentistry to-day is growing too full for one man to successfully master, but it is nevertheless true and must be obvious to all careful observers. It is rapidly dividing into oral surgeons, administrators of anæsthetics, operative and mechanical dentists, and bridge and continuous-gum workers. The one person who attempts all of these will find himself a lamentable failure. The past twelve months have exhibited a marked impulse towards this silent "parting of the ways," and the indications are that specialism will be a still more marked feature of dentistry in succeeding years.

The progress of dental literature has kept pace with the mental activity in other directions. The year has given to dentistry valuable publications, and has shown an advance in periodical work. This promises to be still more apparent in the near future.

The International Dental Journal has, we think, shared in the general upward impulse. The retrospect of this work affords gratification, in that it is felt that the high standard set for its government has been maintained, and that the matter published has been of a positive scientific value, and indicates that undercurrent to which we before alluded. It is anticipated that still greater results will be apparent now that it has happily developed beyond the experimental stage.

The close of the year, therefore, offers every encouragement for renewed energy and enlarged aspirations. We have no fear that the influence of the past will be lost. The impetus has been given slowly, silently, but surely, and we are confident that a constantly- and ever-increasing development will show marked results for the New Year.

